# Immune Cells in Cancer Therapy and Drug Delivery

**DOI:** 10.1155/2016/5230219

**Published:** 2016-04-24

**Authors:** Ceren Eyileten, Kinga Majchrzak, Zofia Pilch, Katarzyna Tonecka, Joanna Mucha, Bartlomiej Taciak, Katarzyna Ulewicz, Katarzyna Witt, Alberto Boffi, Magdalena Krol, Tomasz P. Rygiel

**Affiliations:** ^1^Department of Physiological Sciences, Faculty of Veterinary Medicine, Warsaw University of Life Sciences, Nowoursynowska 166, 02-787 Warsaw, Poland; ^2^Department of Immunology, Center for Biostructure Research, Medical University of Warsaw, Banacha 1a, 02-089 Warsaw, Poland; ^3^Cellis Sp. z o.o., Gen. Zajaczka 28, 01-510 Warsaw, Poland; ^4^Department of Biochemical Sciences, Sapienza University of Rome, Piazzale Aldo Moro 5, 00185 Rome, Italy

## Abstract

Recent studies indicate the critical role of tumour associated macrophages, tumour associated neutrophils, dendritic cells, T lymphocytes, and natural killer cells in tumourigenesis. These cells can have a significant impact on the tumour microenvironment via their production of cytokines and chemokines. Additionally, products secreted from all these cells have defined specific roles in regulating tumour cell proliferation, angiogenesis, and metastasis. They act in a protumour capacity* in vivo* as evidenced by the recent studies indicating that macrophages, T cells, and neutrophils may be manipulated to exhibit cytotoxic activity against tumours. Therefore therapy targeting these cells may be promising, or they may constitute drug or anticancer particles delivery systems to the tumours. Herein, we discussed all these possibilities that may be used in cancer treatment.

## 1. Introduction

Neoplasm is a systemic disease where cancer cells act as a leading devil supported by other cells in the surrounding environment. Particularly, inappropriate activation of the stroma and distant metastasis induced by its components can potentiate and accelerate tumour progression towards a high rate of disease mortality [1]. This microenvironment may differ depending on the tumour type and tissue of origin. It is usually composed of the fibroblasts, adipocytes, pericytes, endothelial cells, and immune cells (macrophages, neutrophils, lymphocytes, dendritic cells, natural killers, or myeloid-derived suppressor cells) which contribute to the tumour progression.

## 2. Macrophages as Drug Targets

Tumour associated macrophages (TAMs), which reside in the tumour mass, play central role in this intratumoural dialog [2]. Cells of the monocyte-macrophage lineage are characterized by considerable diversity and plasticity. In response to various signals, macrophages may undergo classical or alternative activation called M1 or M2, respectively. However, currently it is known that macrophages do not form stable subsets which could be clearly distinguished among each other but respond to a combination of factors present in the tissue which can change their phenotype towards many subforms. Therefore, it is recommended to characterize macrophages by the cytokine used for the activation instead of naming them M1 or M2 [3]. Classically activated macrophages (e.g., LPS activated) have the potential to exhibit antitumour activity whereas alternatively activated (e.g., IL-4 activated) macrophages (called in tumours TAMs) generally have low tumouricidal activity but they promote tissue remodeling and angiogenesis [4]. Therefore they promote tumour development and its spread to distant sites. However, due to high plasticity of macrophages, this process may be reversible and therefore therapeutically exploitable.

The research concerning macrophages in cancer escalated after Lin et al. showed the role of colony stimulating factor 1 (CSF-1) in tumour development, which is normally required for macrophage development. Number and size of primary tumours in CSF-1 knockout mice were similar to the control mice [5]. However CSF-1 deficient mice had lower macrophage number and decreases in tumour progression and metastatic spread. Furthermore, blocking of the CCL2 (chemokine ligand 2), which is secreted by breast cancer cells, in order to recruit metastasis-associated macrophages from the circulation, slows down the growth of tumour metastases [6]. Johnson & Johnson developed CCL2 blocking antibody named CNTO 888 (carlumab) which shows binding affinity to human CCL2 and therefore it decreases macrophage infiltration at the site of challenge. The CNTO 888 is currently in clinical trials for solid tumours; however it does not show antimetastatic activity when used as a single therapy, neither does it block CCL2-CCR2 axis in prostate cancer [7].

Another approach of antimacrophage therapy is to use CXCR4 inhibitors (which are anti-HIV drugs: AMD3100, AMD1498, ALX40-4C, or T22) [8]. The CXCR4 receptor lies downstream in the Hypoxia Inducible Factor (HIF) pathway and therefore increases macrophage infiltration in the tumour and takes part in angiogenesis and cancer progression. Using a mouse model of breast cancer, Welford et al. showed that one of the compounds mentioned above (AMD3100) reduced macrophage recruitment to the tumours and significantly augmented the antitumour efficacy of combretastatin A4P [9]. These results supported previous findings of Welford et al. that TIE-2^+^ macrophages limit the efficacy of combretastatin.

Lisa Coussens has developed a completely different drug limiting macrophage infiltration to the tumour. This molecule called PLX3397 (provided by Plexxikon) targets CSF-1R and when used together with standard chemotherapy, in mice with aggressive mammary cancer, reduced pulmonary metastases regulated by macrophages. PLX3397 increased the cytotoxic T lymphocyte infiltration which resulted in reduced primary tumour development, decreased pulmonary metastases, and improved overall survival [10]. Our own experiments showed that targeting of CSF-1/CSF-1R axis may be a good therapeutic approach in cancer cells [11]. We showed that* csf-1r* silencing significantly increased apoptosis, decreased proliferation, and decreased migration of canine mammary cancer cells. It also changed growth characteristics of highly invasive cell lines on 3D matrix significantly decreasing the invasive ability of these cells.

**Figure 1:**
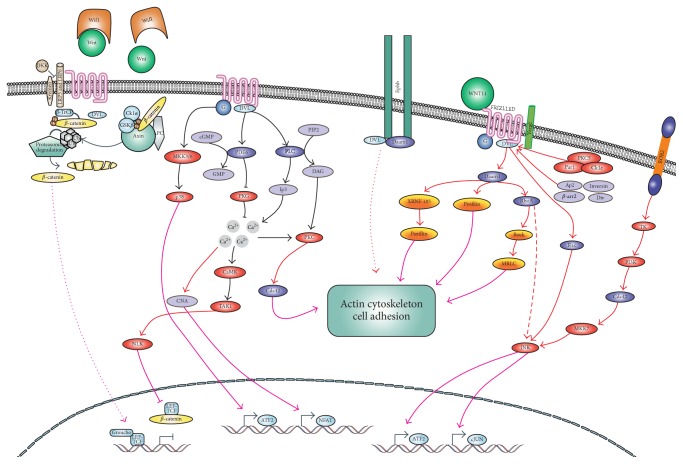
Wnt signaling and macrophages. Wnt signaling pathway is one of the most important pathways regulating cells proliferation, differentiation, polarity, and migration. At least two distinct pathways transduce Wnt signals: canonical Wnt/*β*-catenin pathway and the *β*-catenin independent noncanonical Wnt pathway (Wnt/Ca^2+^ signaling and Wnt/planar cell polarity (PCP) signaling). Wnt/*β*-catenin signaling pathway is upregulated in many cancers. Lack of *β*-catenin degradation and its nuclear accumulation is an evidence of activated Wnt/*β*-catenin pathway. *β*-catenin acts in the nucleus as a transcription factor increasing cancer proliferation and survival. Activation of Wnt/PCP signaling during development results in cytoskeleton remodeling (by Rho, Ras, and JNK) promoting cell movement. Calcium dependent Wnt increases the motility of various cell types by regulating the formation of lamellipodia. It also increases expression of vimentin and therefore induces an epithelial-mesenchymal transition, the crucial step in metastasis. Macrophages infiltrated to tumour mass inhibit cell proliferation as effect of inhibition of Wnt/*β*-catenin pathway. However canonical and noncanonical Wnt pathways work on the principle of antagonism, so macrophages inhibiting *β*-catenin pathway activate noncanonical Wnt pathway and lead to cytoskeleton remodeling in cancer cells and facilitate their motility.

We also showed that manipulating within Wnt signaling may be also a good therapeutic approach. For the first time, tumour associated macrophages mediated a “switch” between canonical and noncanonical Wnt signaling pathway in cancer cells [2]. This “switch” leads to inhibition of canonical Wnt pathway by noncanonical Wnt pathway. Macrophages secrete proteins that inhibit canonical Wnt pathway and decrease cancer cell proliferation and survival. However, the side effect of their function,* ipso facto*, is the activation of noncanonical Wnt pathway. The noncanonical Wnt pathway promotes epithelial-mesenchymal transition, cancer cell motility, invasiveness, and as a consequence their metastasis (Figure 1). Thus, some kind of modulation of macrophage effect on cancer cell by inhibiting of noncanonical Wnt pathway in tumour cells could create a novel and very attractive approach in cancer treatment. This approach would be interesting because it does not impair anticancer activity of macrophages (decrease of tumour growth and survival by inhibition of canonical Wnt pathway) but it may reduce their side effects (metastasis by inhibition of noncanonical Wnt signaling).

An interesting approach to use macrophages in cancer therapy was proposed by Tseng et al. [12] who used macrophages to increase T cell immune response. Exploiting anti-CD47 antibody-mediated phagocytosis of cancer cells by macrophages, the authors showed increased priming of CD8^+^ cells but decreased priming of CD4^+^ cells. It resulted in reduction of regulatory T cells level and decreased tumour mass in animals. Therefore, the conclusion is that anti-CD47 antibody treatment not only enabled macrophage phagocytosis of cancer cells but could also initiate an antitumour cytotoxic T cell immune response.

Pahl et al. [13] tried to modify the macrophage phenotype and thus to induce their antitumour response. By treating M1-like macrophages with liposomal muramyl tripeptide (L-MTP-PE) and IFN-*γ*, authors observed a cytotoxic effect of activated macrophages towards osteosarcoma. This effect was also observed when osteosarcoma cells were treated with supernatants of activated macrophages, suggesting not only direct phagocytosis but also involvement of soluble factors in the cytotoxic effect upon macrophage activation. Whereas stimulation of M2-like macrophages, with LPS and IFN-*γ*, did not drastically change their low antitumour activity, M2 macrophages, stimulated by IL-10 alone, had no impact on tumour growth. However, Pahl et al. demonstrated that IL-10-polarized macrophages had high cytotoxicity towards osteosarcoma cell lines in the presence of anti-EGFR antibodies, which induced antibody-dependent tumour cell phagocytosis. These data suggest the possible benefits of modifying macrophage function based on its subtype within the tumour.

### 2.1. Macrophage Transfer

After the development of technology to generate macrophages* in vitro* from blood monocytes, clinical trials in cancer patients have proven the safety of infusing autologous macrophages activated by interferon-gamma or lipopolysaccharide. Adoptive transfer of host cells may be able to correct defective generation of competent immune cells in patients with cancer [14]. Since the safety of M1-activated macrophages therapy has been proven, several studies using macrophages as a delivery system have been published. Griffiths et al. [15] demonstrated the potential of macrophage use as a cell-based delivery system for gene dependent enzyme prodrug therapy. Macrophage-mediated delivery of the CYP2B6 gene under the constitutive CMV promoter resulted in tumour cell killing in the presence of the prodrug, cyclophosphamide.

In 2011 Muthana et al. [16] showed a novel system that used infiltration of classically activated macrophages, transduced with hypoxia-regulated oncolytic adenovirus in which proliferation was restricted to prostate tumour cells. Using this system, authors markedly inhibited tumour growth and reduced metastases. The same group successfully tested this system after chemotherapy or irradiation during increased tumour infiltration by macrophages [17]. Using this method, authors were able to significantly increase life-span of tumour-bearing mouse as compared to control.

Seo et al. [18] established genetically engineered stable macrophages of RAW264.7 cell line and used them to deliver the prodrug-activating enzyme to the lung melanomas. Animals were treated with inactive prodrug which underwent activation in macrophages. The therapy reduced tumour weights and numbers of melanoma foci.

Firstly, these data indicate that macrophages can constitute targets of anticancer therapy; however the disadvantage of this method is reduction of their positive and physiological activity in live organism. Therefore modulation of their activity seems to represent more appropriate approach. Secondly, macrophages can be exploited as carriers in a gene therapy or as carriers of enzymes activating prodrugs. Macrophages infiltrate diseased tissue and may respond to the hypoxic microenvironment by expression of a therapeutic gene or enzyme. However, the use of viruses in this kind of approach might create unpredictable risk, not only to the treated individuals but also to the population as a whole. The clinical application of oncolytic viruses should be regulated by specific guidelines at international levels. Furthermore, because of the biological limitations of animal models, safety of their preclinical testing should be widely discussed. Cancer is a disease demanding aggressive approaches. However, the balance of risk and benefit must always be of prime consideration, not only for the patients but also for the whole population [19].

## 3. Neutrophils

Neutrophils are traditionally considered as the first line of host defense against invading pathogens [20]. They kill invading pathogens by releasing activating cytokines along with reactive oxygen species (ROS). Despite the role in host defense, they have impact on tumour development being part of its microenvironment [21] and they also have powerful antitumoural effect under certain circumstances [22]. However, the role of neutrophils in the tumour microenvironment is not yet fully understood. Recent studies demonstrated that tumour associated neutrophils (TANs) can promote tumour development, increase metastasis, and enhance angiogenesis [23]. On the other hand some studies showed that neutrophils can be cytotoxic for the tumour cells* in vitro *and* in vivo *[24, 25]. Similarly to macrophages the first are called TAN-2; the latter are called TAN-1.

### 3.1. Neutrophils in Tumour Progression

Neutrophils were shown to have angiogenic effect through the release of multiple factors. TANs have influence on tumour cells via oncostatin M which is a cytokine belonging to interleukin-6 (IL-6) family [26]. In the experiment of TANs coculture with breast cancer cells, this cytokine induced angiogenesis and invasiveness of the latter [27]. Additionally, ROS released by neutrophils may play an important role in tumour progression. Güngör et al. demonstrated that major neutrophilic oxidant hypochlorous acid (HOCl) induced three different types of DNA damage and mutagenicity* in vitro* in human alveolar epithelial lung cells [28]. It was also reported that the proteinase of neutrophil elastase (NE) produced by TANs promotes tumour cell proliferation in both human and mouse lung adenocarcinomas [29]. Another potential direct effect of neutrophils on tumour progression is secretion of matrix metalloproteinase-9 (MMP-9) enzymes [30]. Bekes et al. demonstrated that highly metastatic human fibrosarcoma and prostate cancer cells recruit neutrophils to primary tumours, which increased angiogenesis and intravasation of cancer cells due to secretion of MMP-9. In their study, inhibition of neutrophil influx by interleukin-8 (IL-8) neutralization decreased tumour angiogenesis and intravasation [31]. In addition, it was shown that secretion of MMP-9 by neutrophils prevents apoptosis of tumour cells and induces carcinogenesis [32]. More recently a publication by Bald et al. showed the involvement of neutrophils in induction of migration and metastasis of melanoma cells [33]. In that study, UV-damaged epidermal keratinocytes released nuclear proteins (high mobility group box 1, HMGB1) that caused recruitment and activation of neutrophils. Activated neutrophils produced TNF*α* that increased motility of melanoma cells.

### 3.2. Neutrophils in Therapy

The first reports of antitumoural effect of neutrophils were published in 1970; Bubeník et al. and Godleski et al. showed neutrophil activity against human bladder tumours and rat mammary gland carcinosarcoma, respectively [34, 35]. In 1972 Pickaver et al. [36] described the first direct evidence of the cytotoxic effects of neutrophils on tumour cells. They demonstrated that rat neutrophils collected from the peritoneum and incubated with syngenic tumour cells were able to kill them. Neutrophils produce proteases, ROS, and defensins [37] that can directly damage targeted cells [37, 38]. Dallegri et al. showed apoptosis and necrosis of tumour cell due to increased secretion of HOCl by neutrophils [39]. Moreover, the cytotoxic effects of neutrophils on tumour cells can be increased via target-specific antibodies [40, 41] interacting with the Fc*γ* receptors on the surface of neutrophils via their Fc tail [42] inducing antibody-dependent cellular cytotoxicity. Repp et al. showed that neutrophils obtained from patients treated with recombinant human G-CSF expressed Fc*γ*RI receptor which is a high affinity receptor for IgG [43]. Two years later the same group found that neutrophils from patients treated with recombinant human G-CSF are more effective in inducing antibody-dependent cellular cytotoxicity against glioblastoma, squamous cell, and ovarian and breast carcinoma in contrast to the neutrophils from healthy, untreated donors [44]. Another study showed that the concurrent administration of G-CSF and rituximab (a chimeric antibody against the CD20 antigen on normal and malignant B cells) increased the survival rates of mice with non-Hodgkin's lymphoma. The experiments conducted* in vitro* and* in vivo* demonstrated the role of neutrophils stimulated by G-CSF in enhancing the biological antitumour activity of rituximab [45]. Another approach to achieve the antitumoural effect of neutrophils is to change the immunity of tumours. For example, Cavallo et al. transduced mouse mammary adenocarcinoma cell line (TSA) with the IL-2 genes inducing local inflammatory reactions. TSA-IL-2 cells caused neutrophil infiltration to the tumour mass [46]. Similarly, using IL-10-expressing mouse mammary adenocarcinoma model, it was demonstrated that neutrophils play the key role in the early rejection of the tumour [47]. To determine the biological importance of IL-8 which is a strong chemoattractant for neutrophils [48], Schaider et al. examined melanoma cells from primary and metastatic lesions. These cells, when transduced to produce low levels of IL-8, showed impaired growth* in vivo *due to massive neutrophil infiltration [49]. Similarly, ovarian cancer cells, transduced with the IL-8 human and the murine MIP-1a genes, showed impaired tumourigenicity when injected into nude mice. That was accompanied by the massive neutrophil infiltration in the tumour injection site [50].

Another effective approach to increase number of tumour infiltrating neutrophils is to use live bacteria or certain bacterial products. For example,* Mycobacterium bovis* [51],* Corynebacterium parvum* [52],* Clostridium novyi* [53],* Salmonella typhimurium* [54], and* Salmonella choleraesuis* [55] induced neutrophil infiltrations to the tumour microenvironment. Lee et al. administered* S. choleraesuis* to the mouse with orthotopic hepatocellular carcinoma in order to stimulate a potent inflammatory response. It caused reduced intratumoural microvessel density, increased infiltration of neutrophils, induced cancer cell death, and significantly prolonged survival [55]. Since the 1970s* Mycobacterium bovis* bacillus Calmette-Guérin (BCG) vaccine has been commonly used as an adjuvant treatment for bladder cancer after surgery. Just after BCG administration the massive neutrophil infiltration occurs in the bladder [56]. Suttmann et al. supported that neutrophils are compulsory for efficient BCG immunotherapy of bladder cancer and local immune responses [57]. Since then, several other studies also confirmed that BCG-stimulated neutrophils are highly effective in immunotherapy for bladder cancer [58, 59]. Furthermore, Jinesh et al. [60] showed that RT4v6 bladder cancer cells are resistant to BCG-activated TANs. They demonstrated the critical role of increased TNF-*α* in the anticancer effects of BCG-stimulated neutrophils. Using Smac mimetic compound for neutrophil stimulation they effectively killed bladder cancer cells. Antitumour effect of TANs was also demonstrated by Fridlender et al. [22]. They treated mice with SM16, which is a transforming growth factor-*β* (TGF-*β*) inhibitor, and used monoclonal anti-Ly6G antibody for the systemic depletion of neutrophils. This strategy showed that, after TGF-*β* blockage, TANs can undergo N1 phenotype which produce higher levels of TNF-*α*, MIP-1*α*, NO, and H_2_O_2_ and have antitumourigenic and proinflammatory characteristics. Andzinski et al. [61] showed that IFN type I stimulation induced neutrophils polarization towards antitumour phenotype both in mice and in human.

Jaganjac et al. [62] reported that neutrophil infiltration at the site of W256 carcinoma cells in Sprague-Dawley rats was associated with spontaneous tumour regression. Similarly, injection of Sephadex (a granulocyte attractant) reduced the incidence of the W256 carcinoma cells regression due to neutrophils infiltrating the Sephadex injection site instead of the tumour. As stated before, neutrophil recruitment can have a negative impact on the tumour; however use of proinflammatory stimuli (e.g., bacterial products or neutrophil attractants) leads to neutrophil infiltration accompanied by the classical inflation and antitumour responses.

### 3.3. Neutrophils as Delivery Systems

Neutrophil-based drug delivery was also studied in conjunction with microbial resistance against antibiotics. Wendel et al. showed that neutrophils loaded with chlorhexidine (antibacterial drug) can effectively kill* E. coli* and* F. necrophorum* in the mouse liver [63]. Because neutrophils are continuously recruited to the tumour, further studies may focus on using them as drug delivery systems.

Tumour progression is modified by a wide variety of host myeloid cell types, including neutrophils. Recent studies showed serval mechanisms of protumourigenic effects of neutrophils on tumour cell proliferation, increased metastasis, and enhanced angiogenesis, although some studies demonstrated that proinflammatory polarization of neutrophils via multiple signals results in antitumour effects.

## 4. DC in Cancer Immunotherapy

Dendritic cells (DCs) are at the centre of immune system ability to react against cancer cells. DC-based immunotherapy is a type of a vaccination where tumour antigens are loaded into DCs, followed by administration of these modified DCs to the patient, aiming to stimulate specific T cell immunity against cancer cells. Many improvements in this field have been done but effectiveness of such therapies still awaits the major breakthrough. Successful strategies will require combination of this sophisticated cell-based therapy with other, more blunt approaches.

The principle of DC therapy is to exploit basic ability of DCs to stimulate T cell-based anticancer response. Crucial importance of this process is the ability of DCs to cross-present antigens. This process relies on presentation of exogenous antigens (normally presented on MHC class II) via MHC class I, enabling direct CD8^+^ T cell stimulation. For the successful DC-based immunotherapy three main conditions must be met. Firstly, the activated and antigen-loaded DCs must have immunostimulatory potential. Secondly, effector T cells must be able to unleash cytotoxic activity and lastly tumour cells must be susceptible for the immune attack of effector T cells. Currently there is only one DC-based immunotherapy that has been approved for the treatment of cancer. Sipuleucel-T is a biological used in the treatment of hormone-refractory prostate cancer [64]. The methods consist of* in vitro* stimulation of blood-derived autologous antigen presenting cells (APCs) with GM-CSF and their antigen loading and reinfusion to the patient. However, therapeutic benefit of this protocol seems to be limited, since overall survival of patient with phase III clinical trial was increased by 4.1 months as compared with placebo [65].

Selection of the specific cancer antigen has become a criticalstep in DC vaccine design. An “ideal” cancer antigen should be specific for tumour cells and associated with malignant phenotype. Overexpression of cancer antigen should be restricted to all tumour cells in patients treated for a given tumour type, although the primary feature of cancer antigen, in the context of their therapeutical potential, is ability to induce T cell immunity against tumour with confirmed immunological and clinical relevance [66, 67]. There is a growing list of tumour specific antigens; however only 46 of 75 representative cancer antigens, proposed by the National Cancer Institute, induced T cell response in clinical trials, and 20 of them showed evidence of benefits for patients [66]. On the top of the list of tumour antigens suitable for DC-based cancer therapy is WT1 protein (overexpressed in AML) [67] or highly immunogenic MUC-1 (overexpressed and/or hypoglycosylated in numerous cancer types) [68]. Cancer antigens can be delivered to DCs by pulsing with peptides, proteins, and lysates of apoptotic cancer cells. This exogenous supply of proteins and short peptides used to be the favorite and most common method of antigen loading, which allows peptides to be presented in the context of both MHC class I and MHC class II molecules. Some data favor the use of whole antigen over synthetic short peptides, because there is no need to match the MHC haplotype of the patients [69, 70].

Most of the therapeutic protocols use monocyte-derived DCs (moDCs), which require their differentiation into immature DCs and subsequent maturation to DCs [71]. Recent publications have shown that shortening of the time of differentiation/maturation increases the costimulatory potential of DCs [72] with lowered expression of immunosuppressive PD-L1 molecule [73, 74]. The choice of differentiation protocols is absolutely crucial for maximizing immunostimulatory potential of moDCs. Most of the protocols use IL-4 and GM-CSF [75]. However, this classical method can be improved as shown by replacement of IL-4 with IL-15 or IFN*α* [76, 77]. These unconventional differentiation protocols were shown to improve cytotoxicity against AML cells [77, 78]. Not only differentiation but also activation of moDCs by various cytokine cocktails must be optimal. For that TNF*α*, IL-1*β*, IL-6, and PGE2 were shown to deliver positive effects. However, as with differentiation protocol, this classic protocol can also be improved, for example, by stimulation with cytokines or toll-like receptors (TLRs) agonists [79]. Maturation is also affected by induction of chemokine receptors that facilitate movement into regional lymph nodes (e.g., CCR7) and by the synthesis of cytokines that stimulate T cell differentiation and proliferation (e.g., IL-6, IL-12, or IL-10). TLRs have an essential role in the recognition of and in bridging innate and adaptive immunity. DCs play an important role in activation of immune response against viral infections and can recognize such PAMS as ssRNA by TLR7 and TLR8. Synthetic agonist of TLR7/8 (imiquimod) exerts antiviral and anitumour properties and is marketed for the treatment of external genital warts caused by human papillomavirus [80]. In response to TLR ligands, costimulatory molecules, for example, CD86, CD40, and CD83, are rapidly upregulated and lead to a maturation of DCs, increasing formation of MHC-peptide complexes. Use of TLR agonist was shown not only to stimulate T cell-based cytotoxicity but also to dampen Treg immunosuppression and activation of NK cells [72, 81]. Several TLR ligands [poly(I:C), OK-432, and R848] have been included in clinical trials of DC-based immunotherapies [79].

Another tool to improve immunostimulatory potential of DCs is to modulate expression of stimulatory and inhibitory molecules. Example of this strategy is transfection of OX40L or IL-12 to DCs [82, 83]. Alternatively, shRNA was used to target immunosuppressive molecule as PD-L1 or IL-10 [84, 85]. Because the main problem encountered with anti-CTLA-4 treatment is the resistance of advanced tumours, due to the strong tumour-induced T cell tolerance, effectiveness of DC-based therapy could be improved by combination with immune checkpoint inhibitors that target PD-1/PDL-1 or B7/CTLA-4 pathways [86–88]. Several preclinical tumour models are showing that CTLA-4 blockade in combination with DC vaccination primes immune response and potentiates a specific antitumour response. A single dose of DC vaccine/anti-CTLA-4 inhibits tumour growth in 60% of the challenged mice with EL4 lymphoma cells; moreover the vaccine or CTL-4 blockage administrated alone has no potent antitumour effect [89]. Combination of anti-PD-L1 antibody tumour peptide-pulsed DCs (B16 melanoma) resulted in a higher number of melanoma peptide-specific cytotoxic T cells, unfortunately without significant reduction in tumour growth [90]. Blockade of PD-1 reduced Treg cell numbers attenuating their immunosuppressive activity and also encouraged the ability of DCs to stimulate leukemia antigen-specific T cells [85, 91]. Combinatorial therapy of anti-PD-1mAb pidilizumab and DCs vaccination is currently under phase II clinical trial and when used in patients with AML, remission occurs [92]. Furthermore, clinical trial of PD-L1/2-silenced DC vaccination in combination with donor lymphocyte infusions for the treatment of posttransplant leukemia relapse has also been registered [93]. These data suggest that combination of immune checkpoint inhibitors that target CTLA-4 and PD-1 with DC vaccination enhances the efficacy of T cell immunity.

Another checkpoint pathway that was shown to regulate antitumour immune response axis is CD200-CD200R. Lack of CD200R signaling inhibits outgrowth of an endogenous tumour irrespective of CD200 expression by the tumour cells [94]. Tumour-expressed CD200 suppresses antitumour responses, implying the potential of anti-CD200 antibodies for CD200-expressing cancers [95]. Blockade of CD200-CD200R interaction by antibodies leads to decreased tumour growth in immune competent mice [96]. The CD200 surface molecule is a key mediator of immune escape in AML and CD200 contributes to AML-induced immunosuppression through a multifaceted mode of action, which includes alteration of cytokine profile from TH1 to TH2, induction of Treg cells, and suppression of NK cell function [97–99]. An* in vitro* study of AML showed that abrogation of CD200-CD200R interaction enhances the T cell-stimulatory capacity of DCs [100], whereas inhibition of CD200-CD200R interaction was already investigated in clinical trial phase I/II in patients with relapsing or refractory B cell chronic lymphocytic leukemia or multiple myeloma. The antibody was well tolerated, however, without major therapeutic effects. Interesting way to boost CD200-CD200R blockade is its combination with stimulation of TLR7 pathway, as it was shown that lack of CD200 increases TLR7-dependent immune response [101]. However an adverse effect of CD200-CD200R blockade is also possible as CD200 expression increases with progression of squamous cell carcinoma, suggesting that CD200-expressing tumour cells engage and modulate tumour associated myeloid-derived suppressor cells [102, 103].

Recent years have shown major advances in the field of DC-based immunotherapy; however we still wait for the real change delivered to the patients. Most likely successful use of DC-therapies will depend on the combination with other therapies, not necessarily focused on immunostimulation themselves.

## 5. T Cell-Based Therapy 

Cytotoxic T lymphocytes (CTLs) are the immune effector cells that mostly contribute to cancer rejection. They can recognize specific antigens presented by the APCs with class I major histocompatibility complex (MHC class I) molecules and then can mediate elimination of the cell that they have specifically recognized [104]. Therefore, since 1964 scientists tried to treat established tumours in mice, by the transfer of CTLs. Over time, this led to development of strategy termed adoptive cell therapy (ACT). Transferred T cells exhibit specific antitumour activity in cancer patients. There can be two sources of such T cells: (1) natural host T cells identified in the tumour, the autologous tumour infiltrating lymphocytes (TILs), and (2) T cells from patients' blood that have been genetically engineered* ex vivo* with specific antitumour T cell receptors (TCRs) or chimeric antigen receptors (CARs) [105].

### 5.1. TILs Used in ACT

This strategy is currently the most effective treatment for patients with metastatic melanoma, which is considered as the most immunogenic tumour [106, 107]. The gene aberrations that cause high mutational heterogeneity of melanoma malignancies might be associated with higher probability of the presence of antigen-specific T cells within the tumour [108]. Indeed, melanoma seems to be the only tumour that reproducibly allows obtaining of TILs capable of specific antitumour recognition [107]. After homogenization of a tumour, TILs are cultured for 1-2 weeks* ex vivo* with high dose of interleukin 2 (IL-2). Then, T cells are tested for their antitumour reactivity in coculture assays. These cultures that respond to the tumour antigens (i.e., by production of IFN-*γ*) are activated and expanded to large numbers (1 × 10^11^ cells). After 5-6 weeks T cells are infused back into the patient followed by administration of IL-2, which is the most potent lymphocyte growth factor. Bolus infusion of IL-2 enhances efficacy of ACT, enabling survival and proliferation of adoptively transferred lymphocytes* in vivo* [109]. Further significant improvement of ACT effectiveness using TILs is achieved when the specific preconditioning of cancer patient is applied. This includes lymphodepletion achieved either by chemotherapy (high dose cyclophosphamide and fludarabine) or by total body irradiation (TBI) of nonmyeloablative (2 Gy) or myeloablative (12 Gy) dose [110, 111].

### 5.2. ACT Using Genetically Engineered T Lymphocytes

ACT using genetically modified T cells allows for treatment of other types of cancers, that is, cervical cancer and lymphoma and leukemia and prostate and bile duct cancer and neuroblastoma [109]. It became possible by the introduction of lymphocyte genes encoding conventional T cells receptor (TCR) or chimeric antigen receptor (CAR) [112]. The transduction is based on retroviral or lentiviral vectors or CRISPR technology [113]. Transduced T cell expresses the receptor for specific antigen that can be recognized in MHC independent manner. First target for chimeric antigen receptor was CD19, the molecule present on B cells. CAR therapy was therefore used primarily for the patients with high-risk B cell malignancies; however, by using different targets, use of CARs is being extended to solid tumours. Since the first generation of CAR was used, a lot of improvement in terms of its construction has been made. Second and third generation of this receptor contain not only main domain zeta but also one or more costimulatory domains (CD28, ICOS, and 41BB) that provide complete activation signal for T cells. Thus, transferred T cells are able to proliferate and survive* in vivo* without becoming anergic [114].

All these reports suggest that T cells may play key role in tumour elimination. The treatment of cancer with genetically modified T cells was already demonstrated with both preclinical and clinical studies. However, further researches and new clinical trials are needed to fully understand the antitumour effect of T cells.

## 6. NK Cell-Based Cancer Immunotherapy

Natural killer (NK) cells play a key role in cancer immune-surveillance as they exhibit natural cytotoxicity against many tumour cells even in the absence of preimmunization or stimulation and are virtually able to eradicate malignant cells [115]. As such, NK cell-based immunotherapy holds a great promise for cancer treatment. Thus far, however, the therapeutic potential of NK cell-based immunotherapy has yet to be realized. The major impairment is due to the cancer cell response itself that entails mechanisms to escape NK cell action or induce defective NK cells. Early investigations, using autologous lymphokine-activated NK cells, achieved limited clinical success in cancer patients [116]. Current approaches have thus evolved towards the use of expanded allogeneic NK cells, which are not inhibited by self- histocompatibility antigens like autologous NK cells or stable allogeneic NK cell lines that are more suitable for large-scale production. Alternatively, genetically engineered NK cell lines that are able to express high levels of cytokines, Fc receptors, and/or chimeric tumour antigen receptors have been recently proposed [117]. Progress in understanding NK cell biology and function is, however, needed to foster the development of novel approaches able to address therapeutic NK cells protocols.

### 6.1. Biological Role of NK Cells in Tumour Immune-Surveillance and Therapeutic Perspectives

NK cells comprise 5–15% of circulating lymphocytes and provide a first line of defense against cancer. They display potentially powerful weapons that may provide immediate, short-lived responses by delivering toxic enzymes or releasing cytokines that directly lyse tumour cells or mediate T or B cells immune responses [118]. As previously reviewed deeply by Cheng et al. NK cells are activated by initial recognition of altered receptor patterns on the surface of the target cell, NK cell recognition of tumour cells by inhibitory and activating receptors is a complex phenomenon, and at least three recognition models have been proposed, namely, “missing-self,” “nonself,” and “stress-induced self.” In fact, upon cellular transformation, surface MHC-I expression on tumour cells is often downregulated or eventually not present (“missing-self”), in order to evade recognition by antitumour T cells. Human tumour cells with poor self-MHC-I expression or bearing “altered-self” stress-inducible proteins are thus the preferred NK cell targets for potential therapy. NK cell inhibitory receptors are able to detect the absence of MHC-I expression and mediate cytotoxicity against defective cancer cells [117]. Activation and expansion of NK cells via cytokines such as IL-2, IL-12, IL-15, IL-18, IL-21, and type I IFNs have been studied* ex vivo *[119, 120]. Lang at al. showed the increased activity of NK cells via IL-2 stimulation* in vitro *[119]. After that, Leong et al. demonstrated that preactivation of NK cells with IL-12, IL-15, and IL-18 has shown significantly enhanced antitumour effects [121]. Similarly Kobayashi et al. studied very high doses of IL-15 to observe any meaningful antitumour effects of activated and expanded NK cells* in vitro* and these cells were effective* in vivo *in a lung metastasis mouse model [122].

Therapies designed to induce either a passive or active antitumour response by harnessing the power of NK cells are a most appealing strategy to control tumour development. Despite the multiple properties of NK cells, malignant cells can develop mechanisms to evade immune-surveillance and establish an immune-privileged environment. Some tumour cells may in fact produce immunosuppressive cytokines IL-10 and transforming growth factor-*β* (TGF-*β*), thus impairing the adaptive antitumour immune response, or eventually shift the immune response towards a Th2 response with less antitumour capacity [123]. In order to overcome these limitations, novel generations of genetically modified NK cell lines are being exploited in order to obtain high numbers of functional NK cells that have the potential to survive* in vivo* and are capable of expressing cytokine or overexpressing activating receptors. Retargeting NK cells via chimeric receptors by genetic manipulation approaches has also been proposed to modulate and enhance NK-tumour cell interaction [124, 125]. Clinical trials are currently carried out in haematological malignancies including leukemia and myelodysplastic/proliferative diseases and recently applied also to solid tumours [126].

The design of new strategies, including adjuvant therapies or genetic engineering of NK cells, is currently pursued in order to maximize the cytotoxic potential of NK cells to treat human malignancies.

## 7. Future Perspectives for Using Immune Cells in Cancer Therapy

The role of immune cells in cancer development cannot be underestimated. Thus, prospective therapies targeting these cells may increase effectiveness of cancer treatment. The three most important directions in the development of therapies concentrating around immune cells should be considered. Firstly, there are attempts to decrease the number of immunosuppressive cells, promoting the tumour growth and metastasis, by blocking activity of chemokines recruiting immune cells. Secondly, there are therapies switching off metastasis promoting activities of immune cells, which will modulate their activity by blocking or activating desired functions. Searching for ligands that will enable these changes is of the highest importance. Also genetically engineered, derived from the host, activated immune cells cultured* ex vivo* with knocked-out or knocked-in genes may be utilized as an element of complex therapy. Finally, some of immune cells may be considered as delivery systems transporting antitumour factors directly to the destination place.

Improvement of knowledge about processes taking place in tumour microenvironment will allow creation of specific, personalized therapy. Better understanding of suppressive tumour environment may allow combining therapy, for example, DC or TIL vaccinations with agents tackling immunosuppressive mechanisms (GM-CSF, IFN, IL-2, IL-15, and TNF). It is important to improve knowledge of tumour microenvironment biology, enabling a wider use of recombinant immune cells, cytokines, tumour associated antigens, viruses, and so forth. One of the most important aspects is gathering of information about the escape strategies used by tumours.

Next step will be identification of mechanism(s) used by the tumour of individual patients in order to select the most appropriate approach for each patient to counteract tumour escape. Spreading of tumour immunotherapy will allow using it in earlier stages of the treatment or even in minimal residual disease. It may provide improvement of therapy due to less compromised immune system by chemo- or radiotherapy pretreatment.

## 8. Conclusion

**Figure 2:**
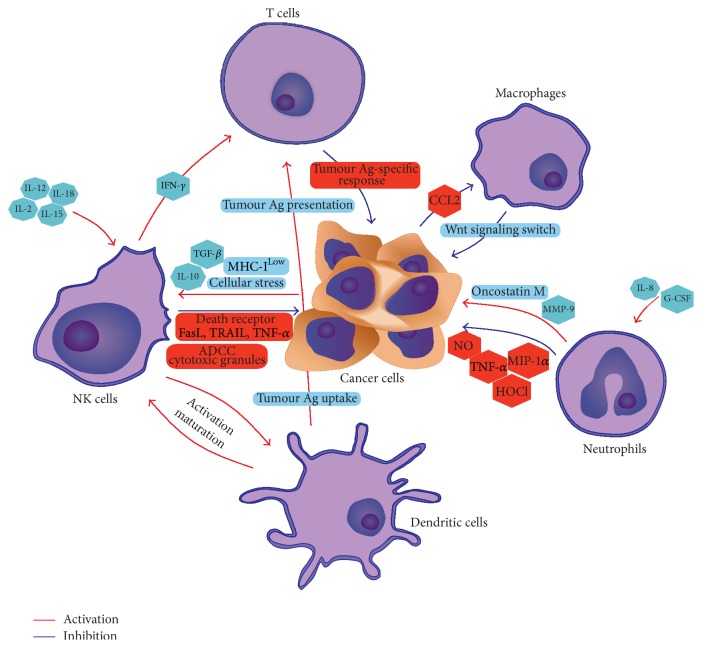
Immune cells in tumour microenvironment. The figure shows the potential roles of immune cells in tumour immunosurveillance. NK cells activated by cancer cells (cellular stress and low expression of MHC-I and IL-10 and TGF-*β*) directly recognize and attack cancer cells through at least four mechanisms: cytoplasmic granule release, death receptor-induced apoptosis, effector molecule production, or ADCC. Interaction of NK cells with DCs leads to improving their antigen uptake and presentation, facilitating the generation of antigen-specific T cells responses. Tumour associated neutrophils secrete oncostatin M inducing angiogenesis and invasiveness of tumour cells. Potential direct effect of neutrophils on tumour progression is secretion of matrix metalloproteinase-9 (MMP-9) enzymes. Inhibition of neutrophil influx by interleukin-8 (IL-8) neutralization can decrease tumour angiogenesis and intravasation. Infiltration of macrophages to tumour microenvironment inhibits canonical Wnt signaling leading to decreased proliferation and survival of cancer cells but as “side effect” noncanonical Wnt signaling is activated inducing metastasis. Ab, antibody; ADCC, antibody-dependent cellular cytotoxicity; DC, dendritic cell; IFN, interferon; and NK, natural killer.

Tumour progression is modified by a wide variety of host cell types, where the key role is played by tumour associated macrophages, T lymphocytes, natural killer cells, tumour associated neutrophils, and dendritic cells. Despite earlier studies showing that these cells might exhibit cytotoxicity towards tumour cells, recent discoveries have indicated that they promote tumour progression by increase of cancer cell proliferation, metastasis, and enhanced angiogenesis. Therefore, they may constitute targets for further anticancer therapy.  Figure 2 summarizes interactions between the immune cells in the tumour microenvironment. However, better understanding of the phenotypic and functional properties of these cells is still required.
